# Mechanically Reinforced Gelatin Hydrogels by Introducing Slidable Supramolecular Cross-Linkers

**DOI:** 10.3390/polym11111787

**Published:** 2019-11-01

**Authors:** Dae Hoon Lee, Atsushi Tamura, Yoshinori Arisaka, Ji-Hun Seo, Nobuhiko Yui

**Affiliations:** 1 Department of Organic Biomaterials, Institute of Biomaterials and Bioengineering, Tokyo Medical and Dental University (TMDU), 2-3-10 Kanda-Surugadai, Chiyoda, Tokyo 101-0062, Japan; lee.org@tmd.ac.jp (D.H.L.); arisaka.org@tmd.ac.jp (Y.A.); yui.org@tmd.ac.jp (N.Y.); 2 Department of Materials Science and Engineering, School of Engineering, Korea University, 145 Anam-ro, Seongbuk-gu, Seoul 02841, Korea; seojh79@korea.ac.kr

**Keywords:** gelatin, polyrotaxane, chemical cross-linking, stretchability, hysteresis loss

## Abstract

Tough mechanical properties are generally required for tissue substitutes used in regeneration of damaged tissue, as these substitutes must be able to withstand the external physical force caused by stretching. Gelatin, a biopolymer derived from collagen, is a biocompatible and cell adhesive material, and is thus widely utilized as a component of biomaterials. However, the application of gelatin hydrogels as a tissue substitute is limited owing to their insufficient mechanical properties. Chemical cross-linking is a promising method to improve the mechanical properties of hydrogels. We examined the potential of the chemical cross-linking of gelatin hydrogels with carboxy-group-modified polyrotaxanes (PRXs), a supramolecular polymer comprising a poly(ethylene glycol) chain threaded into the cavity of α-cyclodextrins (α-CDs), to improve mechanical properties such as stretchability and toughness. Cross-linking gelatin hydrogels with threading α-CDs in PRXs could allow for freely mobile cross-linking points to potentially improve the mechanical properties. Indeed, the stretchability and toughness of gelatin hydrogels cross-linked with PRXs were slightly higher than those of the hydrogels with the conventional chemical cross-linkers 1-ethyl-3-(3-dimethylaminopropyl) carbodiimide (EDC)/*N*-hydroxysuccinimide (NHS). In addition, the hysteresis loss of gelatin hydrogels cross-linked with PRXs after repeated stretching and relaxation cycles in a hydrated state was remarkably improved in comparison with that of conventional cross-linked hydrogels. It is considered that the freely mobile cross-linking points of gelatin hydrogels cross-linked with PRXs attenuates the stress concentration. Accordingly, gelatin hydrogels cross-linked with PRXs would provide excellent mechanical properties as biocompatible tissue substitutes exposed to a continuous external physical force.

## 1. Introduction

Tissue substitutes are utilized to complement the functions of tissues that have been damaged by an innate disease or strong external forces [1]. Hydrogel-type scaffolds show good potential as tissue substitutes because they offer an environment that is approximately similar to the extracellular matrix [2]. However, hydrogel-type scaffolds have limited applications for tissue regeneration owing to their insufficient mechanical properties [3], which is a critical requirement of a tissue substitute to endure the continuous external physical forces on the body. The mechanical properties of hydrogels used for tissue engineering are generally controlled by different factors such as the cross-link density [4,5] and molecular weight distribution [6]. To solve this problem, many researchers have studied strategies for enhancing the mechanical properties of hydrogels with physical or chemical methods [7,8,9,10,11].

Gelatin, a fibrous protein derived from collagen, is a suitable biomaterial for fabricating scaffolds because of its excellent biocompatibility, biodegradability, and non-antigenicity with host tissues [12,13]. The formation of gelatin hydrogels is governed by a temperature-dependent helix-to-coil transition. When the temperature of the gelatin solution is decreased below 35 °C, the disordered peptide chains of gelatin return to a collagen triple-helix structure, which acts as a cross-linked gel junction matrix stabilized by intermolecular hydrogen bonds [14]. In general, even if conventional cross-linkers such as 1-ethyl-3-(3-dimethylaminopropyl) carbodiimide (EDC)/*N*-hydroxysuccinimide (NHS) or glutaraldehyde are used to increase the mechanical properties via chemical cross-linking between lysine and glutamic/aspartic acid residues, it is still difficult to make mechanically suitable gelatin hydrogels for tissue regeneration.

**Figure 1:**
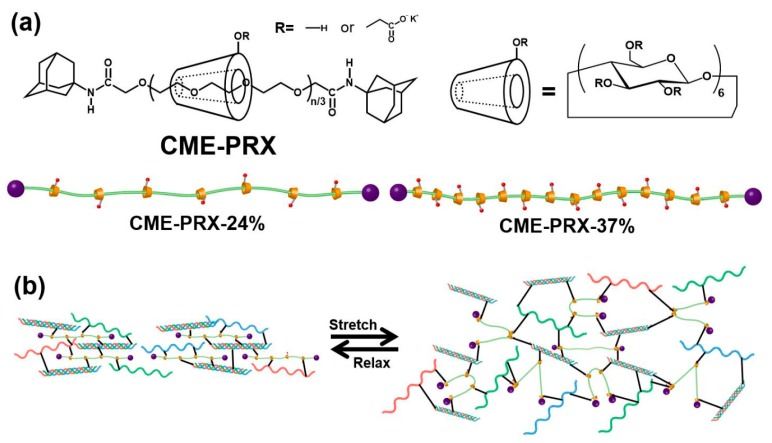
(**a**) Chemical structure of carboxymethyl ether group-modified polyrotaxanes (CME-PRXs); (**b**) Schematic illustration of gelatin hydrogels cross-linked with PRX under a stretch-and-relax environment.

Polyrotaxanes (PRXs) are supermolecules composed of a linear polymer such as poly(ethylene glycol) (PEG) that is threaded into cyclic molecules such as α-cyclodextrins (α-CDs) and capped with bulky molecules (Figure 1a) [15,16]. Since there is no covalent bond between the polymer axle and the cyclic molecules, the threading cyclic molecules in PRXs can freely rotate or move along the polymer axle [17,18]. Owing to their intramolecular mobility property, PRXs have diverse biomedical application potential, such as in regulating cell adhesion and differentiation and for enhancing cell internalization [19,20,21]. Slide-ring gels synthesized by the chemical cross-linking of PRXs show distinct properties from those of conventional physical and chemical hydrogels [22,23]. The cross-linking points in the slide-ring gels can be freely shifted by the movable α-CDs in PRXs along with an axle polymer [24]. When the slide-ring gels are deformed, the stress applied to hydrogels can be dispersed by movement of the cross-linking points without bond breaking, which leads to unique mechanical properties such as high stretchability and toughness [25,26]. For example, hydrogels of poly(*N*-isopropylacrylamide) (PNIPAM) chemically cross-linked with PRXs also show excellent mechanical properties, suggesting that the introduction of movable cross-linking points by PRXs are useful for improving the mechanical properties of various hydrogels. Moreover, slide-ring gels show low hysteresis similar to chemical gels because the cross-link points revert to the original position as soon as the applied strain is released [26]. Ito and co-workers reported that the mechanical properties of slide-ring gels were similar to those of mammalian tissues such as the vessels and skin, suggesting that slide-ring gels can be applied to various biomaterials for tissue regeneration [27].

In this study, gelatin hydrogels chemically cross-linked with PRXs were prepared with the aim of improving their mechanical properties (Figure 1b), which were investigated in comparison to those of gelatin hydrogels cross-linked with EDC/NHS as the conventional method. We hypothesized that mechanically reinforced gelatin hydrogels can be realized when PRXs are chemically cross-linked with gelatin molecules because the cross-linking points can be re-oriented by the mobility of α-CDs in the PRX cross-linkers (Figure 1b).

## 2. Materials and Methods

### 2.1. Materials

Unmodified PRXs composed of α-CDs, PEG (*M*_n_ = 35,000), and 1-adamantyl capping groups were synthesized according to a previous report (see the Supplementary Materials and Figure S1) [28]. Bromoacetic acid, EDC, NHS, and 2,4,6-trinitrobenzenesulfonic acid (TNBS) were obtained from Tokyo Chemical Industry (Tokyo, Japan). Gelatin type B (225 g Bloom) was supplied by Nitta Gelatin (Osaka, Japan).

### 2.2. Synthesis of Carboxymethyl Ether Group-Modified PRXs (CME-PRXs)

CME-PRXs were synthesized according to a previous report (Figure 1a and Figure S1) [29,30]. PRXs (0.5 g, 3.88 × 10^−6^ mol; *M*_n_ = 129,000) were dissolved in 1.49 M KOH solution (25 mL) and stirred for 1 h. To this solution, bromoacetic acid (2.59 g, 1.86 × 10^−2^ mol) was added and stirred at room temperature for 24 h. After the reaction, the solution was placed in a dialysis membrane (molecular weight cut-off of 3500) and dialyzed by distilled water for 48 h. Finally, the solution was freeze-dried to yield CME-PRXs (0.42 g, 69.1% yield).

### 2.3. Characterization of CME-PRXs

^1^H nuclear magnetic resonance (NMR) spectra were recorded in D_2_O at 25 °C using a Bruker Avance III 400 MHz spectrometer (Bruker BioSpin, Rheinstetten, Germany). Fourier transform infrared (FT-IR) spectra were recorded on a Spectrum 100 FT-IR spectrometer (Perkin Elmer, Wellesley, MA, USA). The samples were ground to powder form, which was blended with KBr to prepare pellets for measurements. Aqueous-phase size exclusion chromatography (SEC) measurements were performed using a high-performance liquid chromatography setup consisting of an AU-950 autosampler (Jasco, Tokyo, Japan), PU-980 pump (Jasco, Tokyo, Japan), DG-2080-53 degasser (Jasco, Tokyo, Japan), CO-965 column oven (Jasco, Tokyo, Japan), RI-2031 Plus refractive index detector (Jasco, Tokyo, Japan), and a combination of TSKgel G4000PW_XL_ and G2500PW_XL_ columns (300 mm × 7.8 mm ID; Tosoh, Tokyo, Japan). The sample solutions (10 mg mL^−1^, 50 μm) were injected into the system and eluted with a 100 × 10^−3^ M NaNO_3_ solution at a flow rate of 0.4 mL min^−1^ at 40 °C [30].

### 2.4. Preparation of Gelatin Hydrogels Cross-Linked by CME-PRXs

The gelatin hydrogels cross-linked by CME-PRXs were prepared according to previous reports [31,32,33]. In brief, gelatin powder (100 mg) was dissolved in 0.1 M 2-(*N*-morpholino) ethanesulfonic acid (MES) buffer (1.0 mL) at 40 °C. CME-PRXs, EDC, and NHS were dissolved in 0.1 M MES buffer (1.0 mL) and activated for 10 min at room temperature. Activated CME-PRX solutions were then added to the gelatin solution (1:1 volume ratio) and stirred for 1 min at 40 °C. Finally, the mixture (2 mL) was set in a Teflon mold (30 mm × 60 mm × 1 mm) and gelated at 4 °C overnight. The fabricated gelatin hydrogels were washed in distilled water to remove the byproducts and unreacted EDC/NHS (1 h, four times). The concentration of CME-PRX cross-linkers was first set to 0.5, 1, and 6.35 mg/mL to confirm the optimal condition of mechanical properties in gelatin hydrogels (Table S1).

### 2.5. TNBS Assay

The TNBS assay for gelatin hydrogels was conducted as described previously [34,35]. In brief, dried gelatin hydrogels cross-linked by CME-PRXs (2–4 mg) were immersed and incubated in a mixture of 1 mL of a 0.5% (w/v) TNBS solution (1 mL) and 4% (w/v) NaHCO_3_ (1 mL) for 2 h at 40 °C. After the reaction was complete, 6 M HCl (3 mL) was added to the mixture, and the gelatin hydrogels cross-linked by CME-PRXs were dissolved for 1.5 h at 60 °C. After cooling to room temperature, distilled water (5 mL) was added to the mixtures and the absorbance of the supernatants was measured at 345 nm. The amount of free amino groups in the hydrogels was calculated as follows:

(1) $${\mathrm{Free}\;\mathrm{NH}}_{2}\;\mathrm{groups}=\frac{A\times V}{\varepsilon \times l\times m}$$

where A: absorbance, V: volume (mL), ε: 14,600 (L mol^−1^cm^−1^), *l*: path length (cm), and m: sample weight (mg).

### 2.6. Swelling Test of Gelatin Hydrogels Cross-Linked by CME-PRXs

Gelatin hydrogels cross-linked by CME-PRXs were frozen and freeze-dried. After measuring the weight of the dried gelatin hydrogels (W_D_), they were immersed in phosphate-buffered saline (PBS) for one day at room temperature. Finally, the weight of the swollen gelatin hydrogels (W_S_) was measured, and the swelling ratio was calculated by the following formula:

(2) $$\mathrm{Swelling}\;\mathrm{ratio}\;\left(\%\right)=\;\frac{W_{S}-W_{D}}{W_{D}}\times 100\;\%.$$

### 2.7. Tensile Strength Test

The tensile strength of the gelatin hydrogels cross-linked by CME-PRXs was confirmed by an EZ-Test (EZ-SX 2N, Shimadzu, Kyoto, Japan). The specimens were prepared in a dumbbell shape with a width and thickness of 2 and 1 mm, respectively. To investigate the mechanical properties of the gelatin hydrogels under biological conditions, the specimens were maintained in a hydrated state with PBS. The specimen was 10 mm long. The tensile test was set to a speed of 0.1 mm/s using a 2 N load cell.

### 2.8. Cyclic Tensile Test

The specimens were prepared in a dumbbell shape with the dimensions described above. To investigate the hysteresis loss of the gelatin hydrogels under biological conditions, the specimens were maintained in a hydrated state with PBS. The cyclic stretching test was set to a speed of 0.1 mm/s using a 2 N load cell. The stretching range of the hydrogels was 10 mm and cyclic stretching was performed 20 times.

### 2.9. Statistical Analysis

All data were analyzed by one-way analysis of variance in Excel software. *p* < 0.05 was considered to indicate statistical significance.

## 3. Results and Discussion

### 3.1. Synthesis and Characterization of CME-PRXs

**Figure 2:**
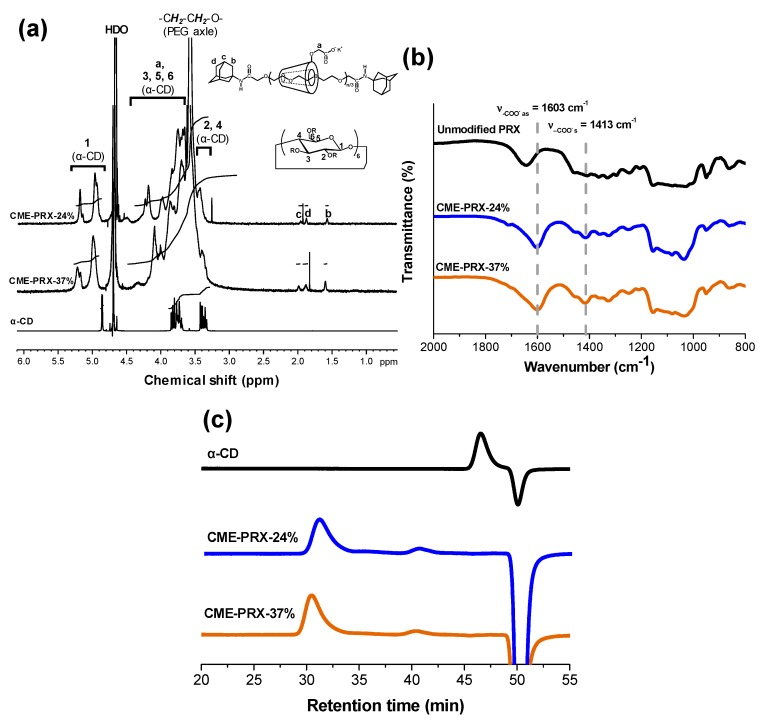
(**a**) ^1^H NMR spectra of CME-PRX-24%, CME-PRX-37%, and α-CD in D_2_O; (**b**) FT-IR spectra of unmodified PRX, CME-PRX-24%, and CME-PRX-37%; (**c**) SEC charts of α-CD, CME-PRX-37%, and CME-PRX-24% in 100 mM NaNO_3_.

In this study, two series of CME-PRXs with different α-CDs threading ratios were prepared to evaluate the effect of the mobility of PRXs (Figure 1a, Table 1, Figures S1 and S2). The introduction of CME groups onto CME-PRXs was characterized by ^1^H NMR and FT-IR (Figure 2). Although the peak of CME moieties (–O–CH_2_–COO^–^ K^+^) should appear between 3.5 and 4.5 ppm in the ^1^H NMR spectrum, this peak was difficult to observe due to overlapping with the peak of α-CDs (Figure 2a). In the FT-IR spectra, asymmetric and symmetric vibration modes of carboxylate anions in CME-PRXs were clearly observed at 1603 and 1413 cm^−1^, respectively (Figure 2b), indicating that the CME groups were successfully introduced in the PRXs as carboxylate anions. The number of CME groups modified on PRXs was calculated by the difference of the integral values of unmodified PRXs and CME-PRXs in the ^1^H NMR spectra (Table 1). Since the number of CME groups in CME-PRX-24% and CME-PRX-37% differed, the number was adjusted to the CME groups per threading α-CDs for effective comparison. To prove whether the CME-PRXs had an interlocked molecular structure composed of α-CDs and PEG, SEC was conducted using an aqueous solution as the mobile phase. The peaks of CME-PRXs were observed at a higher molecular weight region in comparison with those of α-CDs (Figure 2c), suggesting that the CME-PRXs maintained an interlocked polyrotaxane structure based on α-CDs and PEG. In addition, the peak of CME-PRX-37% was shifted to a higher molecular weight region compared to that of CME-PRX-24%, which also supports the finding that large numbers of α-CDs were contained in CME-PRX-37% compared with CME-PRX-24%.

### 3.2. Preparation of CME-PRX Cross-Linked Gelatin Hydrogels

**Figure 3:**
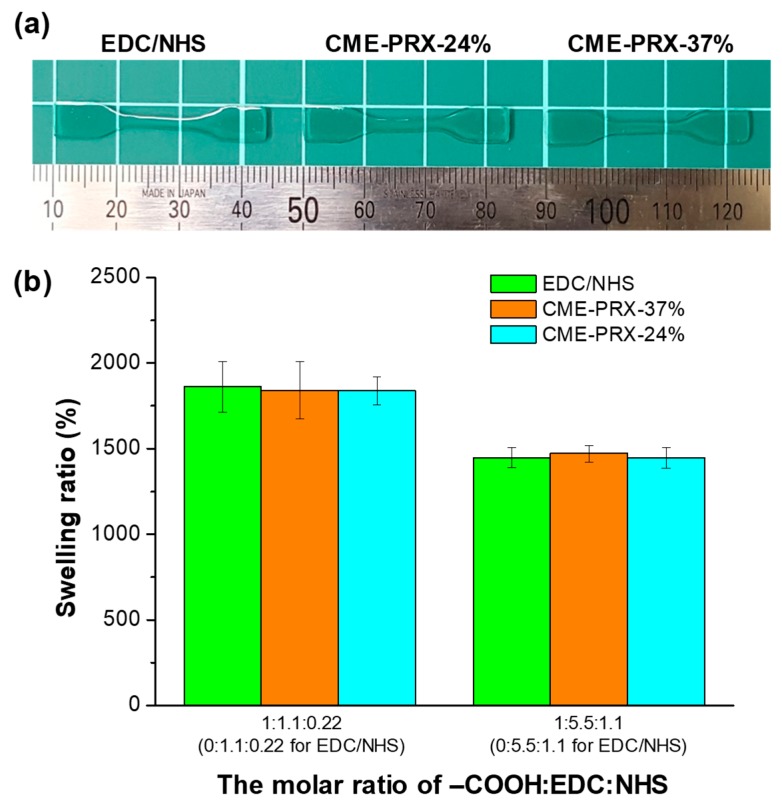
(**a**) Gross images of gelatin hydrogels cross-linked by EDC/NHS, CME-PRX-24%, and CME-PRX-37%; (**b**) Swelling ratio of gelatin hydrogels cross-linked by EDC/NHS, CME-PRX-37%, and CME-PRX-24%.

Gelatin hydrogels cross-linked with CME-PRXs were prepared by the condensation of lysine residues in gelatin and CME groups using EDC and NHS at neutral pH. By varying the molar ratio of the COOH groups in CME-PRXs/EDC/NHS, gelatin hydrogels with different cross-linking degrees were prepared (Table 2 and Table S1). The gelatin hydrogels were obtained at the optimal concentration of CME-PRXs and the molar ratio of COOH groups in CME-PRXs/EDC/NHS (Figure 3a, Table S1). According to these results, the gelatin hydrogels prepared at a CME-PRX concentration of 1 mg/mL were used for further study. Gelatin hydrogels cross-linked with only EDC/NHS were prepared as a control, in which the amounts of EDC and NHS were adjusted to be equivalent to those in the CME-PRXs cross-linked gelatin hydrogels. The cross-linking degree in the gelatin hydrogels was determined by quantifying the amount of unreacted amino groups (lysines) using TNBS. The cross-linking degree in gelatin hydrogels increased with the amount of EDC/NHS. However, the cross-linking degree of the gelatin hydrogels was not significantly affected by the type of cross-linkers used with the same amount of EDC/NHS. To demonstrate the swelling behavior of gelatin hydrogels cross-linked by CME-PRXs, a swelling test was carried out (Figure 3b). The swelling ratios clearly depended on the amounts of EDC/NHS (i.e., the degree of cross-linking), regardless of the type of cross-linkers used.

### 3.3. Mechanical Properties of Gelatin Hydrogels Cross-Linked by CME-PRXs

**Figure 4:**
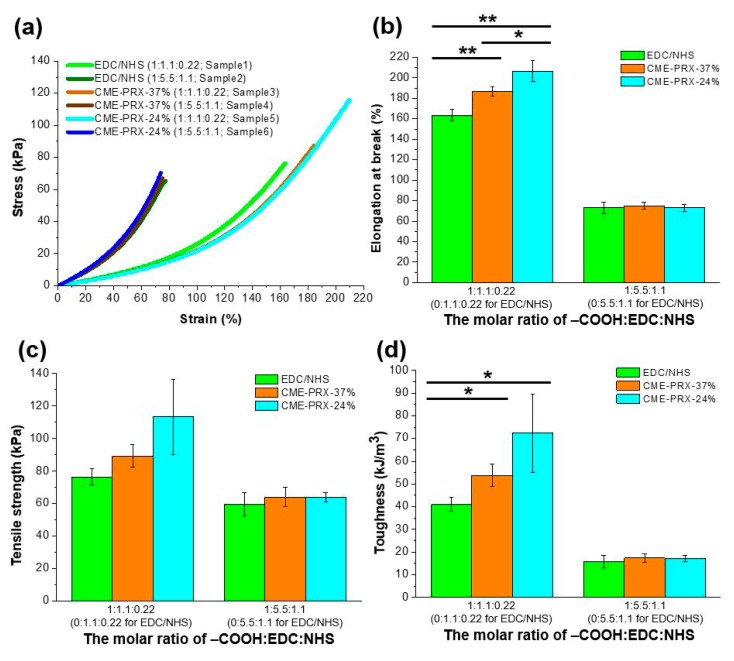
(**a**) Stress–strain curves of gelatin hydrogels cross-linked by CME-PRXs; (**b**) Elongation; (**c**) tensile strength; (**d**) toughness of gelatin hydrogels cross-linked by CME-PRXs (* *p* < 0.05 and ** *p* < 0.01; n = 3).

Figure 4 shows the result of the tensile strength test of gelatin hydrogels in a hydrated state. The tensile strength of gelatin hydrogels cross-linked by EDC/NHS and CME-PRXs exponentially increased with elongation of the hydrogels (Figure 4a). The elongation at break, tensile strength, toughness, and Young’s modulus were determined from the stress–strain curves of the hydrogels. The elongation at break in gelatin hydrogels was increased with small amounts of EDC/NHS (samples 1, 3, and 5 in Table 2). In large amounts of EDC/NHS (samples 2, 4, and 6 in Table 2), gelatin hydrogels cross-linked by different types of cross-linkers showed no significant difference in elongation at around 80% strain (Figure 4b). By contrast, for the groups with small amounts of EDC/NHS (samples 1, 3, and 5 in Table 2), CME-PRX cross-linkers improved the elongation of gelatin hydrogels compared to that obtained using EDC/NHS as the cross-linker. Moreover, there was a significant difference in elongation according to the threaded number of α-CDs in CME-PRX cross-linkers. Tensile strength in gelatin hydrogels was increased with decreasing amounts of EDC/NHS (Figure 4c). Despite the higher tensile strength of gelatin hydrogels cross-linked by CME-PRXs than that of hydrogels cross-linked with EDC/NHS, the differences were not statistically significant. The toughness of the hydrogels can be calculated by the area under the stress–strain curve graph from the tensile strength test [36,37]. Compared to EDC/NHS cross-linkers, CME-PRX cross-linkers significantly improved the toughness of the gelatin hydrogels.

Hydrogels cross-linked with EDC/NHS can be easily broken because the cross-linking points are fixed and applied stress is focused under external forces [27]. However, the cross-linking points in CME-PRX-cross-linked gelatin hydrogels might slide along with the axle polymers in CME-PRXs owing to the movable α-CDs in CME-PRXs. This mobility of α-CDs in CME-PRXs allows for the stress applied in CME-PRX cross-linked gelatin hydrogels to be dispersed, resulting in high elongation, although the tensile strength was comparable to that observed with EDC/NHS cross-linking. If the threaded ratio of α-CDs in CME-PRXs were to decrease, the mobility of α-CDs should also increase. For this reason, the cross-linking points in gelatin hydrogels cross-linked by CME-PRX-24% could be relatively more shifted in comparison with those of CME-PRX-37%, and the elongation of gelatin hydrogels was also improved. Previous studies demonstrated that slide-ring gels showed remarkable stretchability and toughness due to the movable α-CDs in PRXs as cross-linking points [38,39,40]. This is attributed to the greater length of the backbone chain between cross-links compared to that of high-threaded PRXs, and there is low pressure due to the few ring molecules between cross-links [39]. Therefore, it is considered that low-threaded CME-PRXs are suitable for improving the mechanical properties of gelatin hydrogels. However, the mechanical properties of gelatin hydrogels are governed not only by the chemical composition of CME-PRXs but also by the ratio of condensation reagents (i.e., the ratio of EDC/NHS). In the groups with large amounts of EDC/NHS (samples 4 and 6 in Table 2), it is considered that the gelatin molecules might be cross-linked by not only CME-PRXs but also by unreacted excess EDC/NHS. Therefore, no significant differences in elongation and toughness were observed in the groups with large amounts of EDC/NHS.

In previous reports, Young’s modulus in slide-ring gels showed a similar value regardless of the number of threaded cyclic molecules in PRXs, although Young’s modulus increased with a higher cross-linking density [24,41]. In the present study, at the 1% strain point, Young’s modulus was controlled by the amount of EDC/NHS regardless of the type of cross-linker used (Table 2). Note that these Young’s modulus values are comparable to those of previous reports on gelatin hydrogels [42,43]. However, at the fracture point, all types of gelatin hydrogels showed similar Young’s modulus values between 1.24 and 1.84 kPa. When the amounts of EDC/NHS were increased 5fold, Young’s modulus in gelatin hydrogels was increased by approximately 3fold at 1% strain for all types of cross-linkers. However, at the fracture points, there was no significant difference in Young’s modulus regardless of the amount of EDC/NHS used. It was also reported that some characteristics in slide-ring gels are lost at higher moduli in relation with higher cross-linker concentrations [44,45]. In line with these previous reports, we found that the significant differences of Young’s modulus in the gelatin hydrogels disappeared when the cross-linking degree was similar, regardless of the type of cross-linkers used.

### 3.4. Effect of Hysteresis in Gelatin Hydrogels Cross-Linked by CME-PRXs Under Cyclic Stretching

**Figure 5:**
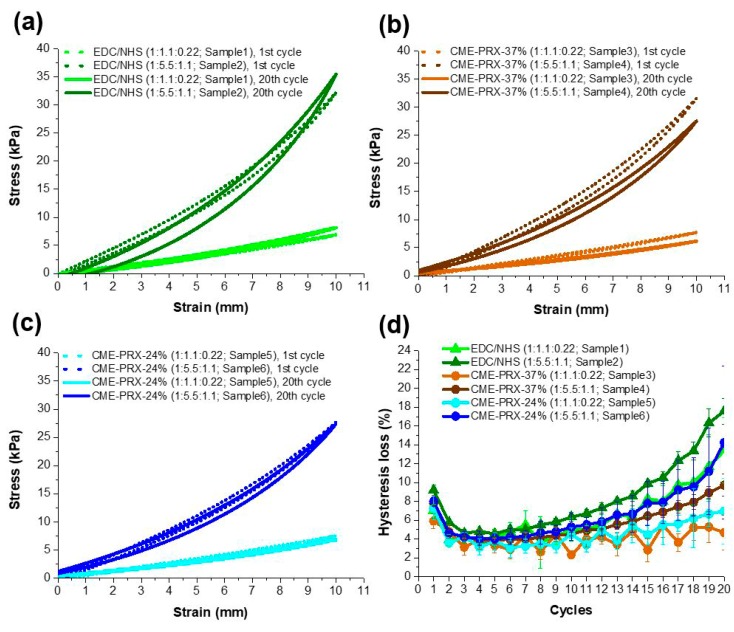
Cyclic tensile test for gelatin hydrogels cross-linked by (**a**) EDC/NHS; (**b**) CME-PRX-37%; (**c**) CME-PRX-24%; (**d**) Hysteresis loss of gelatin hydrogels in each cycle (n = 3).

To investigate the hysteresis loss of gelatin hydrogels cross-linked by CME-PRXs under a cyclic stretching environment, a cyclic tensile test was carried out. In a hydrated state, the gelatin hydrogels cross-linked by CME-PRXs showed lower hysteresis in comparison with those cross-linked with EDC/NHS (Figure 5a–c). All samples showed similar hysteresis loss around 5% until the seventh cycle (Figure 5d). However, from the eighth cycle, the difference of hysteresis loss in each sample gradually increased. Thus, CME-PRX cross-linkers decreased the hysteresis loss of gelatin hydrogels compared to EDC/NHS, and the hysteresis loss decreased with reducing EDC/NHS amounts for activating CME-PRXs. In addition, highly threaded α-CDs in CME-PRXs reduced the hysteresis loss in gelatin hydrogels under cyclic stretching environment. It is considered that the high hysteresis loss in EDC/NHS-cross-linked gelatin hydrogels is caused by higher energy dissipation through the emerging gelatin phase that destroys the new orientation compared to that of the sliding motion of α-CDs [46]. The CME-PRX-24%-cross-linked gelatin hydrogels showed higher hysteresis loss than that of CME-PRX-37%. It is expected that in a hydrated state, the energy from applied stress can be more evenly dispersed due to the high mobility of α-CDs in comparison with the energy dispersion in CME-PRX-37%.

## 4. Conclusions

This study provides a materials-based approach to reinforce not only the stretchability and toughness but also the hysteresis loss of gelatin hydrogels by using PRX cross-linkers. Compared to EDC/NHS-cross-linked gelatin hydrogels, the mobility of α-CDs in CME-PRXs improved the overall mechanical properties of the gelatin hydrogels; however, Young’s modulus of the gelatin hydrogels could only be controlled by the cross-linking degree regardless of the type of cross-linkers used. Furthermore, increasing the hysteresis loss of gelatin hydrogels by cyclic stretching could be controlled with the use of CME-PRX cross-linkers. Controlling the mobility of α-CDs in CME-PRXs could also regulate the stretchability, toughness, and hysteresis loss in gelatin hydrogels. Since hydrogels for biomedical applications must be able to endure continuous external forces in the body, reinforcement of stretchability, toughness, and hysteresis loss in gelatin hydrogels using CME-PRX cross-linkers could be a promising approach for constructing optimal hydrogels for biomedical applications.

## Figures and Tables

**Table 1:** Characterization of carboxymethyl ether group-modified polyrotaxanes (CME-PRXs).

Sample	*M*_n_ of the PEG Axle	Number of Threaded α-CDs onto PRX ^1^	Number of CME Groups on PRX ^2^	*M*_n_^3^
CME-PRX-24%	35,000	96 (24.1 %)	294 (3.06)	157,000
CME-PRX-37%	35,000	147 (37.0 %)	498 (3.39)	227,000

^1^ Determined by ^1^H NMR in D_2_O. The values in parentheses denote the threading percentage of α-CDs in PRX, assuming that one α-CD molecule forms an inclusion complex with two ethylene glycol units in the PEG axle. ^2^ Determined by ^1^H NMR in D_2_O. The values in parentheses denote the average number of CME groups per threaded α-CD in CME-PRXs. ^3^ Calculated based on the chemical composition of the CME-PRXs determined by ^1^H NMR.

**Table 2:** Characterization of gelatin hydrogels cross-linked with CME-PRXs and 1-ethyl-3-(3-dimethylaminopropyl) carbodiimide (EDC)/*N*-hydroxysuccinimide (NHS).

Sample	Cross-Linker Type	Weight Ratio of Gelatin/CME-PRX/EDC/NHS ^1^	Cross-Linking Degree (%)	Young’s Modulus (kPa)	
				1% Strain	Fracture Point
1	EDC/NHS	100:0:0.46:0.06 (0:1.1:0.22)	8.41 ± 4.86	0.11 ± 0.01	1.24 ± 0.20
2	EDC/NHS	100:0:2.31:0.28 (0:5.5:1.1)	18.41 ± 2.09	0.27 ± 0.01	1.79 ± 0.14
3	CME-PRX-37%	100:1:0.46:0.06 (1:1.1:0.22)	9.69 ± 3.66	0.11 ± 0.01	1.31 ± 0.09
4	CME-PRX-37%	100:1:2.31:0.28 (1:5.5:1.1)	17.64 ± 0.51	0.30 ± 0.01	1.84 ± 0.24
5	CME-PRX-24%	100:1.17:0.46:0.06 (1:1.1:0.22)	11.88 ± 3.21	0.11 ± 0.02	1.61 ± 0.30
6	CME-PRX-24%	100:1.17:2.31:0.28 (1:5.5:1.1)	21.69 ± 4.00	0.32 ± 0.02	1.83 ± 0.05

^1^ The ratios in parentheses are the molar ratios of COOH in CME-PRX/EDC/NHS.

## Supplementary Materials

The following are available online at https://www.mdpi.com/2073-4360/11/11/1787/s1, Figure S1: Synthetic scheme of CME-PRXs, Figure S2: SEC charts of α-CD, PEG, PRX-24%, and PRX-37%, Figure S3: Mechanical properties of gelatin hydrogels cross-linked by different amounts of CME-PRX-37% and EDC/NHS, Table S1: Characterization of gelatin hydrogels with different concentration of CME-PRX-37% crosslinkers and amounts of EDC/NHS.
