# Supplementary material for: Mechanically Reinforced Gelatin Hydrogels by Introducing Slidable Supramolecular Cross-Linkers
Source: Polymers (Basel). 2019 Nov 1;11(11):1787. doi: 10.3390/polym11111787 (PMC6918157; doi:10.3390/polym11111787)
Supplement: Supplementary file 1 [file polymers-11-01787-s001.pdf]

Supplementary materials

Mechanically reinforced gelatin hydrogels by introducing slidable supramolecular cross-linkers

Dae Hoon Lee[1], Atsushi Tamura[1,*], Yoshinori Arisaka[1], Ji-Hun Seo[2], and Nobuhiko Yui[1]

[1]Department of Organic Biomaterials, Institute of Biomaterials and Bioengineering, Tokyo Medical and Dental University (TMDU), 2-3-10 Kanda-Surugadai, Chiyoda, Tokyo 101-0062, Japan.

[2]Department of Materials Science and Engineering, School of Engineering, Korea University, 145 Anam-ro, Seongbuk-gu, Seoul 02841, Korea.

*Correspondence: tamura.org@tmd.ac.jp

S1. Materials

Poly(ethylene glycol) (PEG, M_n = 35,000) was obtained from Merck (Darmstadt, Germany). α-Cyclodextrin (α-CD) was obtained from Ensuiko Sugar Refining (Tokyo, Japan). Sodium bromide (NaBr), sodium hypochlorite (NaClO; available chlorine less than 5%), 1*H*-benzotriazol-1-yloxy)tris(dimethylamino)phosphorium hexafluorophosphate (BOP), and *N*,*N*-dimethylformamide (DMF) were obtained from Fujifilm Wako Pure Chemical Corporation (Osaka, Japan). 2,2,6,6-Tetramethylpiperidine 1-oxyl (TEMPO), 1-adamantylamine, and *N*,*N*-diisopropylethylamine (DIPEA) were obtained from Tokyo Chemical Industry (Tokyo, Japan). Other reagents and solvents were obtained from Kanto Chemical (Tokyo, Japan) and Fujifilm Wako Pure Chemical Corporation.

S2. Synthesis of carboxylated PEGs

PEG bearing carboxy groups at the termini (PEG-COOH) were synthesized according to a previous study (Figure S1) [1]. PEG (20 g, 571 μmol) and NaBr (189 mg, 1.84 m mol) were dissolved in distilled water (160 mL), and then NaClO (40 mL) and TEMPO (188 mg, 1.2 mmol) were successively added to the reaction mixture. The pH of the solution was adjusted to 11 by adding 1 M NaOH solution, and the solution was stirred for 90 min at room temperature. The oxidative reaction of PEG was quenched by adding ethanol, and the pH of the solution was adjusted to 2 by adding HCl. The polymer was then extracted by CH_2Cl_2, and the solution was poured into diethyl ether to reprecipitate the polymer. Finally, the reprecipitated polymer was collected by filtration and dried under reduced pressure to yield

PEG-COOH (18.7 g, 93.3% yield). ^{1}H NMR (400 MHz, $CDCl_3$) δ = 3.62 (m, -O-C***H*_2**-C***H*_2**- of PEG axle), 4.13 (s, -OC***H*_2**-C(=O)-).

S3. Synthesis of adamantyl group-capped polyrotaxanes (PRXs)

PRXs composed of α-CD as a cyclic molecule, PEG as an axle polymer, and adamantyl groups as stopper molecules were synthesized according to a previous study (Figure S1) [1]. PEG-COOH (2.5 g, 714 μmol) and α-CDs (5 g, 5.14 mmol) were dissolved in distilled water (24.5 mL) and the mixture was stirred for 24 h at room temperature. The precipitate was collected by centrifugation (8,500 rpm, 3 min) and freeze-dried to obtain a pseudopolyrotaxane (6.02 g). Subsequently, 1-adamantylamine (389 mg, 2.57 mmol), BOP (1.14 g, 2.57 mμmol), and DIPEA (512 μL, 3 m mol) were dissolved in DMF (50.2 mL), and the solution was combined with pseudopolyrotaxane. The mixture was stirred for 24 h at room temperature. After the reaction, the precipitate was collected by centrifugation (8,500 rpm, 3 min), dissolved in a small aliquot of dimethyl sulfoxide (DMSO), reprecipitated in distilled water, and collected by centrifugation (8,500 rpm, 3 min). The reprecipitation process was repeated four times to completely remove the free α-CD and unreacted reagents. The recovered precipitate was freeze-dried to obtain PRX (2.96 g, 32.1% yield based on recovered PEG mol%). The number of threading α-CDs in the PRX was determined from the ^{1}H NMR spectrum. ^{1}H NMR (400 MHz, NaOD/D_2O) δ=1.61 (m, adamantyl group), 1.91 (m, adamantyl group), 2.00 (m, adamantly group), 3.13-4.07 (m, -O-C***H*_2**-C***H*_2**- of PEG axle, H_2, H_3, H_4, H_5, and H_6 protons of α-CD), and 4.94 (m, H_1 proton of α-CD).

PRXs were also characterized by size-exclusion chromatography (SEC) measurements using a Prominence-i LC-2030 Plus system (Shimadzu, Kyoto, Japan) equipped with an RID-20A refractive index detector (Shimadzu) and a combination of TSKgel α-4000 and α-2500 columns (300 mm length, 7.8 mm internal diameter) (Tosoh, Tokyo, Japan) (Figure S2). Sample solutions were injected into the system and were eluted with DMSO containing 10 mM LiBr at a flow rate of 0.35 mL/min at 60 °C.

S4. Preparation of gelatin hydrogels with different amounts of CME-PRX-37% and EDC/NHS.

To verify the optimal conditions for preparing the gelatin hydrogels cross-linked by CME-PRXs, gelatin hydrogels with different amounts of CME-PRX-37% and EDC/NHS were prepared. In brief, the gelatin powder (100 mg) was dissolved in 0.1 M 2-(*N*-morpholino)ethanesulfonic acid (MES) buffer (1.0 mL) at 40 °C. CME-PRXs, EDC, and NHS were dissolved in 0.1 M MES buffer (1.0 mL) and activated for 10 min at room temperature. Activated CME-PRX solutions were then placed in the gelatin solution at a 1:1 volume ratio and stirred for 1 min at 40 °C. The gelatin hydrogels were then maintained at 37 °C to confirm the formation of the gelatin hydrogels cross-linked by CME-PRX-37%.

Stable hydrogels were obtained at optimal CME-PRX-37%:EDC:NHS ratios. In addition, the stretchability and toughness of the gelatin hydrogels cross-linked by CME-PRX-37% were optimal when the hydrogels were prepared at a CME-PRX-37% concentration of 1 mg/mL. By contrast, the stretchability and toughness of the gelatin hydrogels prepared at other CME-PRX-37% concentrations were low, which was probably due to excessive or ineffective cross-linking.

S5. References

[1] Araki, J.; Zhao, C.; Ito, K. Efficient production of polyrotaxanes from α-cyclodextrin and poly(ethylene glycol). *Macromolecules* **2005**, *38*, 7524–7527.

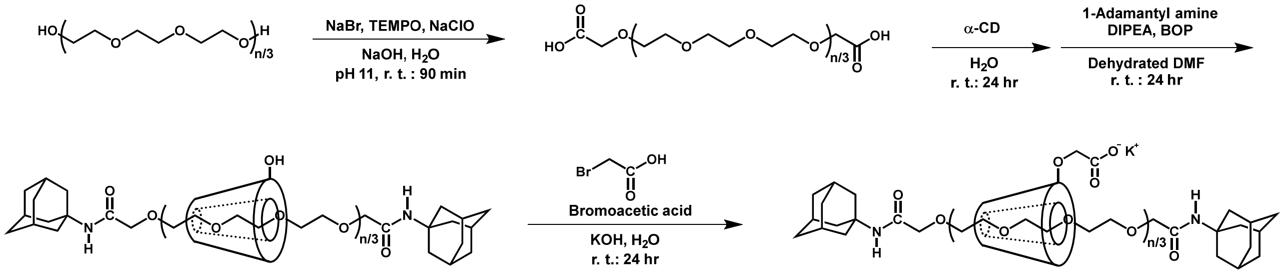

Figure S1. Synthetic scheme of CME-PRXs

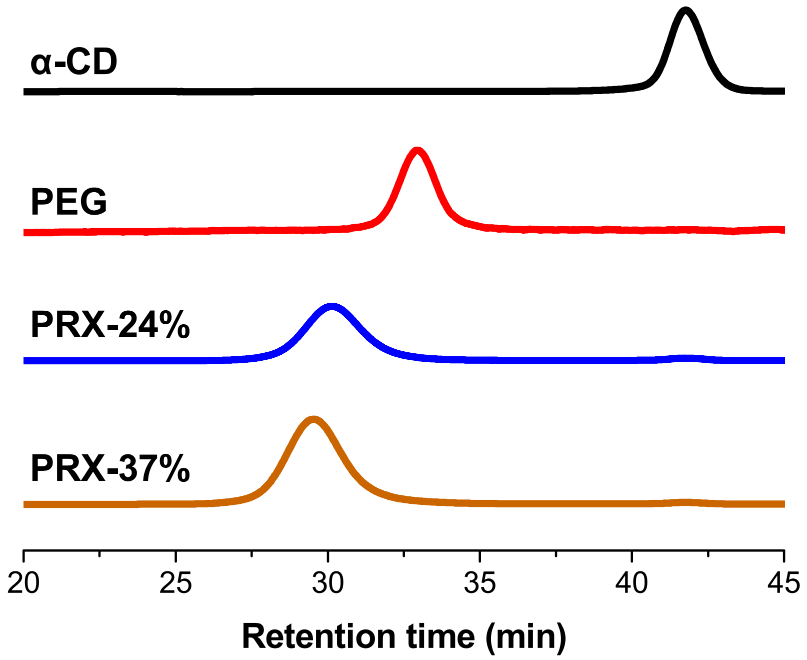

Figure S2. SEC charts of α-CD, PEG (M_n = 35,000), PRX-24%, and PRX-37% in DMSO containing 10 mM LiBr at 60 °C.

Table S1. Characterization of gelatin hydrogels with different concentration of CME-PRX-37% cross-linkers and amounts of EDC/NHS.

Code	Concentration of CME-PRX-37% (mg/mL)	Weight ratio of gelatin:CME-PRX-37%:EDC:NHS[1]	Cross-linking degree (%)	Gel formation at 37 °C
1	0.5	100:0.5:0.02:0.003 (1:0.11:0.02)	5.14 ± 1.43	-
2	0.5	100:0.5:0.12:0.01 (1:0.55:0.11)	5.30 ± 1.97	-
3	0.5	100:0.5:0.23:0.03 (1:1.1:0.22)	8.42 ± 1.67	-
4	0.5	100:0.5:1.16:0.14 (1:5.5:1.1)	13.36 ± 1.03	+
5	1	100:1:0.05:0.005 (1:0.11:0.02)	3.30 ± 1.67	-
6	1	100:1:0.23:0.03 (1:0.55:0.11)	5.89 ± 4.16	-
7	1	100:1:0.46:0.06 (1:1.1:0.22)	9.69 ± 3.66	+
8	1	100:1:2.31:0.28 (1:5.5:1.1)	17.64 ± 0.51	+
9	6.35	100:6.35:0.29:0.03 (1:0.11:0.02)	7.80 ± 0.72	-
10	6.35	100:6.35:1.47:0.18 (1:0.55:0.11)	11.46 ± 4.17	+
11	6.35	100:6.35:2.94:0.35 (1:1.1:0.22)	17.75 ± 0.55	+
12	6.35	100:6.35:14.7:1.76 (1:5.5:1.1)	32.03 ± 3.38	+

[1] The ratios in parentheses are the molar ratios of COOH in CME-PRX-37%:EDC:NHS.

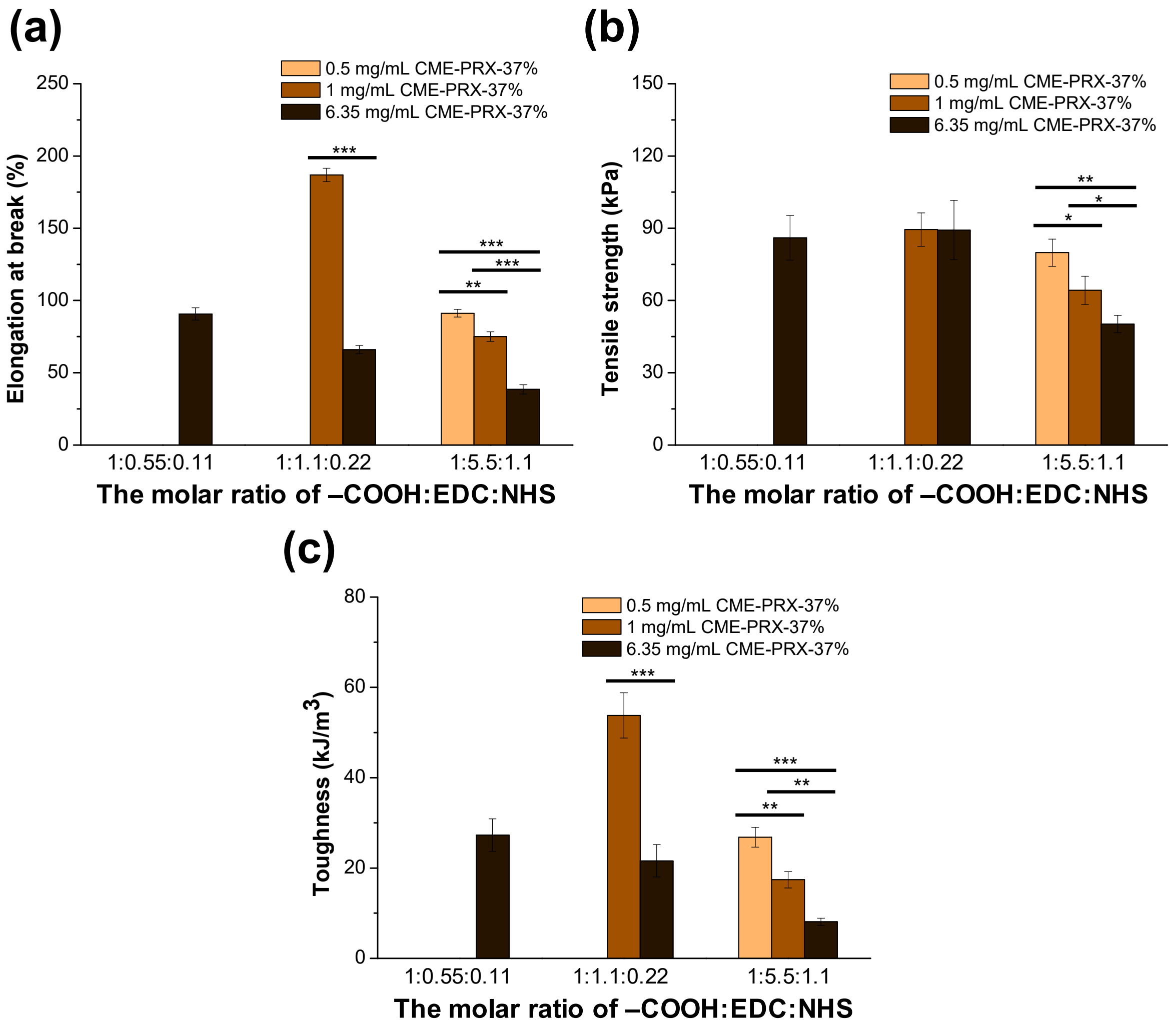

Figure S3. (a) Elongation, (b) tensile strength, and (c) toughness of gelatin hydrogels cross-linked by different amounts of CME-PRX-37% and EDC/NHS.
